# An Account of the Success with Which the Method of Uniting Parts by the First Intention Has Been Adopted in the Radical Cure of the Hydrocele

**Published:** 1787

**Authors:** Thomas Tomlinson

**Affiliations:** Surgeon to the General Hospital at Birmingham


II. *An Account of the Succeſs with which the Method of uniting Parts by the firſt Intention has been adopted in the radical Cure of the Hydrocele. Communicated in a Letter to Dr.* Simmons, *by Mr.* Thomas Tomlinſon, *Surgeon to the General Hoſpital at Birmingham.*

WILLIAM Wathen, a labouring man, about fifty years of age, was admitted into the General Hoſpital on March the 4th, 1786, for the cure of a hydrocele, which had been of about twelve months continuance.

Some

Some time previous to the occurrence of this cafe, it had been fuggefted to me by Mr. Mynors (an eminent furgeon of this town), that the cure might be very much facilitated and forwarded if an union of the parts could be brought on by the firft intention or adhefive inflammation.

This idea carrying full conviction along with it, I did not hefitate a moment about making the experiment, efpecially after having confidered how much lefs pain and confinement would arife from this mode of treatment than from that which is now in moft general ufe.

On the 6th of March I began the operation, by making an incifion the whole length of the fide of the fcrotum affected, cautioufly diffecting down to the tunica vaginalis, through which I made an opening at its upper or fuperior part, and after having introduced my finger, enlarged the aperture down to the moft pendent part of the tumour, by taking out an oval piece of the tunic, in order that the lips of the wound might not be prevented from coming into clofe contact. This being done, and the tefticle examined and found perfectly found, the edges of the integuments were brought together, and retained in their proper fituation situation by means of sutures. To make all secure, pieces of adhesive plaster were applied, and over the whole a roller, of about two fingers in breadth, and three yards in length, was adjusted, being passed once round the body to prevent its slipping off.

The patient was then put to bed, and a pillow filled with bran placed under the scrotum, which I recommend as far preferable to a suspensory bandage.

March 7. He did not complain of much pain arising from the operation; but being rather feverish, a saline draught, with vin. antimonial, was ordered to be taken every three or four hours.

8th. The feverish symptoms were increased; the pain was inconsiderable; and there was very little discharge through the roller. The saline draughts were continued.

9th. The fever was much the same as yesterday. The dressings being removed, I found a general adhesion had taken place, with as little inflammation as could possibly be expected; no more indeed than seemed necessary to produce the desired effect.

10th. The fever was much less than during the preceding day. The sutures were removed, and every thing presented the most pleasing appearance; the discharge being inconsiderable.

11th. The patient was almost entirely free from fever and every uneasy sensation. Being rather costive, a clyster was administered.

12th. He continued to do well: and there was little or no discharge, except at the places from whence the sutures had been removed.

21st. The wound being quite healed, the patient was discharged cured.

It was my intention to have transmitted this case to you at an earlier period: but, reflecting, that objections might be started to the adoption of this mode of practice, from the authority of a single case, I resolved to wait, until I should have an opportunity of seeing more fully the success of this plan, and whether there was any probability of the disease returning.

I have now a particular satisfaction in being able to say, that since the treatment of the above case, I have seen two others conducted in the same manner, with similar success; one of which fell under my own immediate care, and the other, under that of my worthy colleague, Mr. Vaux. In both cases, the febrile symptoms were remarkably mild, and the cure of one one was completed in fifteen days, and of the other in fixteen.

Upon relating to Mr. Mynors the above particulars, and telling him my defign of publifhing them, he authorifed me to fay, in addition thereto, that a cafe of hydrocele, of nine years ftanding, containing about a pint and a half of fluid, in a man of fixty-feven years of age, had, within thefe fix months, fallen under his care; when, upon conducting the method of cure upon the above principles, it was completed in the fpace of three weeks; notwithftanding in that cafe, the tunica vaginalis was extremely thickened, from the long continuance of the difeafe.

*Birmingham,*

March 6, 1786.

